# Spinocerebellar ataxia: Functional analysis of the stomatognathic system

**DOI:** 10.4317/medoral.22839

**Published:** 2019-03

**Authors:** Bruno Ferreira, Marcelo Palinkas, Ligia Gonçalves, Gabriel da Silva, Veridiana Arnoni, Isabela Regalo, Paulo Vasconcelos, Wilson-Marques Júnior, Jaime Hallak, Simone Regalo, Selma Siéssere

**Affiliations:** 1 DDS, Professor. School of Dentistry of Ribeirão Preto, University of São Paulo Department of Morphology, Physiology and Basic Pathology. Rehabilitation and Ribeirão Preto Medical School, University of São Paulo, São Paulo, Brazil; 2 DDS, PhD, Professor. School of Dentistry of Ribeirão Preto, University of São Paulo Department of Morphology, Physiology and Basic Pathology. National Institute and Technology - Translational Medicine (INCT.TM). Postgraduate of the Faculty Anhanguera, Ribeirão Preto, São Paulo, Brazil; 3 DDS, PhD. School of Dentistry of Ribeirão Preto, University of São Paulo Department of Morphology, Physiology and Basic Pathology, São Paulo, Brazil; 4 DDS, PhD, Professor. School of Dentistry of Ribeirão Preto, University of São Paulo Department of Morphology, Physiology and Basic Pathology. Rehabilitation and Ribeirão Preto Medical School, University of São Paulo, São Paulo, Brazil; 5 DDS. School of Dentistry of Ribeirão Preto, University of São Paulo Department of Morphology, Physiology and Basic Pathology, São Paulo, Brazil; 6 DDS, PhD, Professor. Rehabilitation and Ribeirão Preto Medical School, University of São Paulo, São Paulo, Brazil; 7 DDS, PhD, Professor. National Institute and Technology - Translational Medicine (INCT.TM). Rehabilitation and Ribeirão Preto Medical School, University of São Paulo, São Paulo, Brazil; 8 DDS, PhD, Professor. School of Dentistry of Ribeirão Preto, University of São Paulo Department of Morphology, Physiology and Basic Pathology. National Institute and Technology - Translational Medicine (INCT.TM), São Paulo, Brazil

## Abstract

**Background:**

Neurodegenerative diseases that affect the cerebellum, especially in elderly individuals, cause impairment of motor coordination and quality of life. The presente study evaluated the electromyographic activity and thickness of the right and left masseter and temporal muscles, and the maximum molar bite force of individuals with spinocerebellar ataxia.

**Material and Methods:**

Twenty-eight individuals were divided into two groups: those with (n=14) and without (n=14) spinocerebellar ataxia. Data on the masticatory muscles obtained from the electromyographic activity (resting, right and left laterality and protrusion), muscle thickness (maximal voluntary contraction and tensile strength) and maximum bite force (right and left) were tabulated and descriptive analysis using Student’s t-test (*P* ≤ 0.05).

**Results:**

In the comparison between groups, greater electromyographic activity was demonstrated for individuals with spinocerebellar ataxia, with a statistically significant difference in protrusion and laterality for the temporal muscles (*P* = 0.05). There was no statistically significant difference between the groups for masticatory muscles thickness in the conditions evaluated. For maximum molar bite force, the group with spinocerebellar ataxia showed lower bite force (*P* ≤ 0.05).

**Conclusions:**

The data obtained suggest that spinocerebellar ataxia promotes functional reduction in the stomatognathic system, mainly affecting the electromyographic activity and bite force, hindering chewing, with a resultant alteration of nutritional intake and a decrease of quality of life.

** Key words:**Spinocerebellar ataxia, electromyography, muscle thickness, bite force, masseter muscle, temporal muscle.

## Introduction

Neurodegenerative diseases that affect the cerebellum are responsible for several bodily changes that are identified through the involvement of neurological pathways (1,2). Among the neurological disorders that individuals with cerebellar lesion show are ataxias, which is a heterogeneous group of systemic alterations associated with reduction in the quality of life (3,4).

Among the numerous forms of ataxias, spinocerebellar ataxia is defined as a disease that progressively affects the spinocerebellar region with initial clinical manifestations of impaired balance and motor coordination (5,6). The clinical picture is very variable and can include dysmetria, dysdiadochokinesia, and tremors in the appendicular skeleton, signs of pyramidal diseases, cognitive dysfunction, epilepsy, peripheral neuropathies, dementias, parkinsonian syndrome, dystonia, and involuntary movements (7,8).

Individuals with neurological disorders often have dysfunctions related to swallowing and chewing, but little is known, with scientific evidence, what happened with stomatognathic functions (6).

Changes in the stomatognathic system may appear in individuals with clinical signs and symptoms of spinocerebellar ataxia caused by dysphagia, myoclonus, and dysarthria (9,10). These clinical alterations reduce masticatory function, favoring dehydration, malnutrition, tracheal aspiration, reduction of autonomy (11,12).

Considering that individuals with spinocerebellar ataxia show changes in swallowing and chewing, all knowledge related to the stomatognathic system will have clinical applicability.

Our results are relevant to the field of health care, justifying the importance of this study in the construction of new diagnostic and treatment strategies for individuals with spinocerebellar ataxias. The hypothesis of this study is that individuals with spinocerebellar ataxia show decreases bite force and masseter and temporalis muscles thickness, with increased of electromyographic activity.

Therefore, the aimed this study was analyze the electromyographic activity and masseter and temporalis muscles thickness, as well as the maximum molar bite force of individuals with spinocerebellar ataxia and to compare the results to data obtained from individuals without the disease.

## Material and Methods

-Sample 

The experimental protocol was approved by the Human Research Ethics Committee of the Ribeirão Preto School of Dentistry, University of São Paulo, Brazil (number 13073313.9.0000.5419) in compliance with Resolution CNS 466/2012, which regulates all Brazilian clinical research. All individuals signed the informed consent form.

This research is a prospective observational case-control study. From a total of 38 individuals (after considering the inclusion and exclusion criteria) 28 individuals with spinocerebellar ataxia were selected. All these patients had previously been previously diagnosed. The diagnosis of the disease was performed by a specialist neurologist in the area, through clinical examinations carried out at the Ribeirão Preto Medical School of the University of São Paulo, Department of Neurosciences and Behavioral Sciences. Some patients, after the diagnosis, followed treatment in the Basic Health Unit and the Hospital of Bebedouro, which has a specialized center for care and research in the area of ataxia.

A post hoc sample size calculation was conducted considering a level of α=0.05, a power of 99% for the main outcome electromyographic activity in rest condition (mean of the right temporal muscle, AG= 0.22 [0.06] and CG= 0.11 [0.02]), effect size of 2.45. The minimal sample size obtained was 28 volunteers (14 for each group). Sample size calculation was performed with the G*Power 3.0.10 software.

All subjects in the sample were examined by a dentist, a specialist in occlusion, and classified as individuals with normal occlusion, as class I of angle; as having the presence of a complete permanent dentition (except upper and lower third molars), lacking postural and/or cognitive alterations; and with an absence of a temporomandibular disorder (Research Diagnostic Criteria for Temporomandibular Disorders -RDC-TMD).

The exclusion criteria for the group with spinocerebellar ataxia were: requirement of ventilatory assistance; probe for power supply; and lack of clinical diagnosis issued by neurologist, as well as voluntary discontinuation of the proposed treatment for the disease.

The individuals were divided into two groups: those with spinocerebellar ataxia (average age 44.0 ± 3.7 years; average BMI 24.28 ± 0.89 Kg/m2; n = 14) and those without the disease (average age 43.3 ± 3.8 years; average BMI 24.51 ± 0.80 Kg/m2; n = 14). Subjects in the non-disease group were matched with the spinocerebellar ataxia group by age, gender, and body mass index (BMI). There was no statistically significant difference in the comparison of age variables (*P* = 0.98) e IMC (*P* = 0.85) between the two groups.

-EMG Analysis

The MyoSystem Br1 electromyograph (DataHominis Technology Ltda, Uberlândia, Minas Gerais, Brazil) with surface active electrodes (Datahominis Ltda., Model DHT-easd; two 10-mm long × 2-mm wide silver chloride bars 10-mm apart) with an input impedance of 1010 Ω/6, pf, a bias current input of ±2 nA, a common-mode rejection ratio of 110 dB at 60 Hz, and a gain equal to 20× was used to measure the electromyographic activity (microvolts / seconds) of the masseter and temporal muscles.

The conditions evaluated for the mandibular position were: rest (4 s), dental tightening during maximal voluntary contraction (4 s), protrusion (4 s), and right and left laterality (4 s for each condition).

The surface electrodes were positioned according to the recommendations of the SENIAM project (Surface EMG for Non-Invasive Assessment of muscles) (13). The processed and filtered EMG signal was used to derive amplitude values obtained by the square root of the mean (RMS) calculation.

The patients were instructed before electromyography to sit in a chair with their feet flat on the floor and their palms resting on their thighs; to keep their bodies relaxed and to breathe slowly. The horizontal plane of Frankfurt was kept parallel to the ground.

-Analysis of Muscle Thickness

SonoSite Titan ultrasound equipment was used (SonoSite, Inc., Bothell, Washington, USA) with a 10 MHz linear transducer to analyze the thickness of the ultrasound images of the masseter and temporalis muscles, in the mandibular rest position and during the maximal voluntary contraction. This was done after the patients performed three exams for each experimental condition, with an interval of 2 minutes between each measurement. The transverse positioning of the linear transducer was performed on the temporalis muscle belly located in the region of the temporal fossa, about 1.0 to 1.5 cm back and upward from the lateral commissure of the eyelids on both sides. For the masseter muscle, the transducer was positioned approximately 1.5 to 2.0 cm above the angle of the mandible toward the zygomatic arch (14).

The averages of the values obtained from these three ultrasound images were calculated for each experimental condition. Muscle thickness was assessed after the electromyographic examination, and the surface electrodes were then removed for ultrasound examination.

-Analysis of the Maximum Molar Bite Force

The equipment used to evaluate the maximum first molar bite force (right and left) was a digital dynamometer (Kratos, Cotia, São Paulo, Brazil), with a capacity of 980.66 N, adapted to the conditions of the mouth.

The maximum bite force was obtained in the region of the first permanent molars with the patient sitting in a chair and the occlusal plane parallel to the floor (15). Three measurements were obtained on each side of the arcade, alternating the right and the left side, with a two-minute interval between each maximum bite (16). The maximum molar bite force was obtained by the highest value of the three forces exerted on each side of the dental arch (17,18).

-Method Error

The error of the method was calculated using Dahlberg’s formula (19). Measurements of electromyographic activity, maximal bite force, and muscle thickness were calculated using the records of ten individuals during two different sessions, with a one-week interval between the sessions. The data showed small variations in the measurements between the first and second sessions in electromyographic activity (3.74%), muscle thickness (4.38%) and bite force (5.21%).

The reliability of the intra-rater variability was calculated by the intra-class correlation coefficient (ICC). The reliability was considered acceptable in the electromyographic activity (ICC = 0,936; lower bond 0.898 and upper bond 0.960), muscle thickness (ICC = 0,999; lower bond 0.996 and upper bond 1.000) and molar bite force (ICC = 0,928; lower bond 0.105 and upper bond 0.995).

-Statistical Analysis

The electromyographic data was normalized by dental tightening in maximum voluntary contraction. Statistical analyzes were performed in the SPSS program (Statistical Package for the Social Sciences) version 21.0 for Windows (IBM, SPSS, Chicago, USA). Descriptive analysis (average and standard deviation) and normality test (normal distribution) were performed for each variable. Values were compared using Student’s test (*P* ≤ 0.05).

## Results

 Table 1 shows the normalized RMS values for each muscle studied at each clinical condition (rest, protrusion, right and left laterality) for both groups.

**Table 1:**
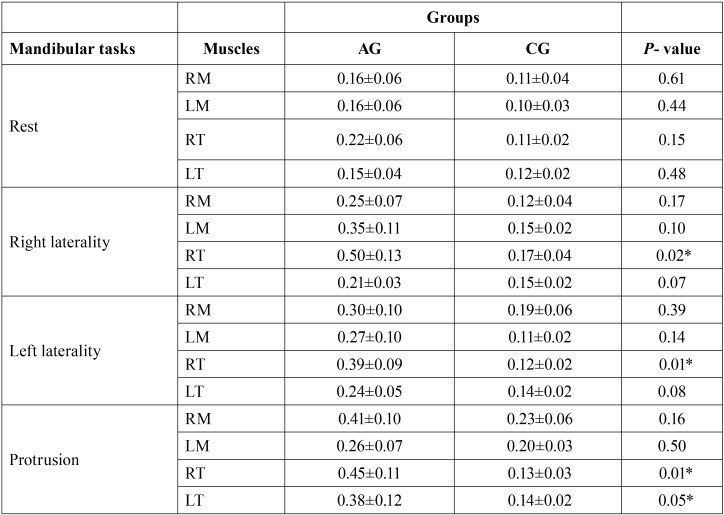
Mean (±SD) and statistical significance (*P* ≤ 0.05) * of the normalized electromyographic data of the right masseter (RM), left masseter (LM), right temporal (RT), left temporal (LT) muscles for spinocerebellar ataxia group (AG) and control group (CG) in mandibular task.

During the clinical condition of protrusion, there was statistically significant difference in the electromyographic activity for the right temporal (*P* = 0.01) and left temporal (*P* = 0.05). Greater myoelectrical activity was observed in the group with spinocerebellar ataxia.

In the laterality, greater activation for right temporal muscle (*P* = 0.02) was found during the condition of right laterality and greater activation of the right temporal muscle (*P* = 0.01) during the condition of left laterality for the group with spinocerebellar ataxia. The group with spinocerebellar ataxia presented the highest values in the EMG activity of the masseter and temporalis muscles in the mandibular tasks.

The mean thickness values (in centimeters) of the masseter and temporalis muscles at rest and maximal voluntary contraction between groups are shown in  Table 2. There were no statistically significant differences among the groups, although the spinocerebellar ataxia group showed clinically greater muscle thickness.

**Table 2:**
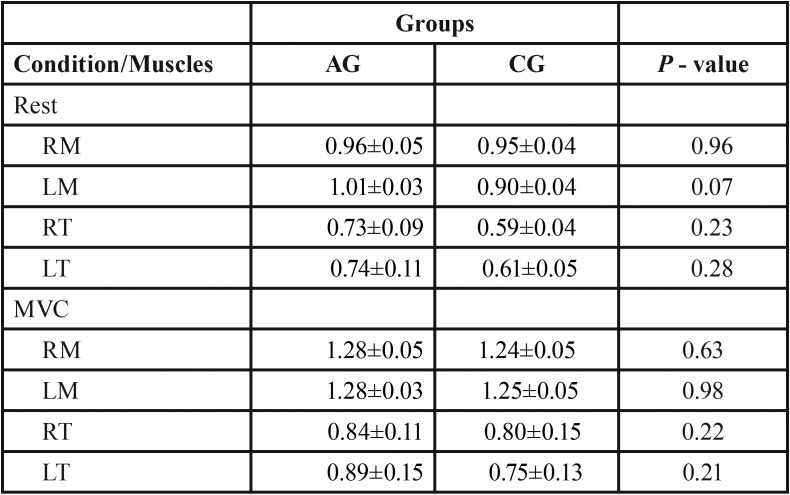
Mean (±SD) and statistical significance (*P* ≤ 0.05) * of the muscle thickness (cm) data of the right masseter (RM), left masseter (LM), right temporal (RT), left temporal (LT) muscles for spinocerebellar ataxia group (AG) and control group (CG) in the mandibular rest and maximum voluntary contraction (MVC).

 Table 3 shows the mean values of the maximum first molar bite force (right and left). Maximum bite force is measured in N. There was statistically significant difference between the analyzed groups for the right molar region (*P* = 0.001) and left molar region (*P* = 0.04). Lower maximum molar bite force was found in the spinocerebellar ataxia group.

**Table 3:**
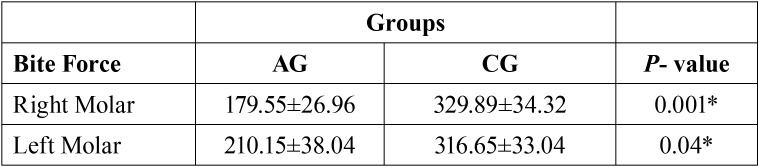
Mean (±SD) and statistical significance (*P* ≤ 0.05) * of maximum molar bite force (N) for spinocerebellar ataxia group (AG) and control group (CG) (right and left molar regions).

## Discussion

The knowledge of the functional changes in the organism due to the presence of spinocerebellar ataxia and its repercussion in the stomatognathic system is fundamental in the evaluation and treatment of patients who develop this disease. This mainly affects the clinical professional, in regard to issues patients have with oral rehabilitation, the use of dental implants, bite force, masticatory efficiency, and others problems.

 The functional alterations of the masseter and temporalis muscles activity observed in this study were measured by internationally recognized monitoring techniques, such as surface electromyography, during several postural conditions of the mandible. We corroborate studies of Silva *et al.* (20) and Oliveira *et al.* (21) who used the same methodology, but with groups of individuals with chronic degenerative diseases.

Our results demonstrated that individuals with spinocerebellar ataxia showed greater electromyographic activity in all postural conditions of the mandible when compared to the group without the disease. This may be a result of increased duration and amplitude of motor unit action potentials associated with reduced muscle recruitment density in individuals with neurodegenerative diseases (22). Probably, these muscular alterations occurred because individuals with ataxia showed a change in the mandibular reflex, originating from injuries in the brainstem itself, which causes a loss in the control of the motor neurons of the trigeminal nerve and of the muscles innervated by this segment (23).

In the protrusion condition, the spinocerebellar ataxia group demonstrated greater electromyographic activity of the temporal muscles compared to the masseters. Contrary to what has been observed in patients with ataxia, de Oliveira *et al.* (24) observed that in the protrusion condition of healthy individuals a greater activity of the masseter muscles occurs than in the temporal ones.

The neurological system needs to be intact to avoid errors in motor action, and when compromised it damage pathologies in muscle activation, influencing the motor coordination of individuals (25). One hypothesis that may also explain a greater activation of the temporal muscles would be the presence of psychological stress, since there is integration between the emotional system and social behavior within motor pathways. This integration promotes facial expressions that trigger increased electrical signals of the temporal muscles (17). However, the levels of psychological stress were not evaluated in this study.

In the right laterality condition, both groups showed greater electromyographic activity of the contralateral, ipsilateral, temporal and masseter muscles to the mandibular movement, corroborating the studies of da Silva *et al.* (26). In the left laterality condition, the spinocerebellar ataxia group showed greater muscle activation of the right temporal (contralateral muscle) compared to the left (ipsilateral) temporal one, showing, once again, an electromyographic activity not considered standard for this clinical condition (27).

One hypothesis that may explain the changes in electromyographic activity in the right and left laterality is that due to the pathophysiology of ataxia itself it becomes difficult to perform activities that require advanced motor coordination. This could be due to the pathological changes in the cerebellum, which impair the synchrony and precision of movements (28).

Individuals who develop spinocerebellar ataxia show progressive neuronal loss in cortical areas and there is a dense connection between the spinocerebellar region and the frontal cortical motor area. Thus, lesions in these pathways can trigger changes in muscle control during the movement execution phase, as shown by electromyographic activity (29).

The measurement of the thickness of the temporal muscles and masseters from the conditions of mandibular rest and dental tightening in maximum voluntary contraction is an essential method of analysis of the stomatognathic system. We clinically experimental demonstrated that the group with spinocerebellar ataxia showed greater thickness in all muscles evaluated for both experimental conditions, though without statistically significant differences. Our results corroborate those of Verhagen *et al.* (30) who reported an increase in the thickness of upper and lower limb muscles in subjects with ataxia, with no statistically significant differences. The authors further described the increase in the echo intensity of the image of these individuals that are credited to the possible morphological alterations of the musculature.

Thus, we believe that ataxia alters the muscular architecture of these patients since the increased demand of muscular activity can trigger biochemical factors that lead to muscular hypertrophy in individuals afflicted with the disease (29). We are not aware of recent studies evaluating the morphology of muscle tissue, nor has there been an analysis of the muscular thickness in the stomatognathic system in individuals with ataxia, to compare to the data obtained in this study.

The individuals in this study were submitted to an evaluation of the maximum molar bite force, a technique capable of objectively analyzing the masticatory function (31). The maximum molar bite force was analyzed in this study by positioning the dynamometer on the region of the first permanent molar (17). Our results demonstrate that the group with spinocerebellar ataxia showed a reduction of the maximum molar bite force with statistically significant values for the right and left sides. These findings corroborated those of Verhagen *et al.* (30) who observed a muscle strength reduction in individuals with ataxia . These results are expected because with the progression of the disease it is expected that a greater degeneration of the central nervous system occurs, increasing the clinical signs of the subject. This is something we observed in this study because we found a significant reduction of the functionality of the stomatognathic system, as evidenced by the decrease of bite force (25).

However, no studies comparing maximal molar bite strength of individuals with ataxia and without the disease were found, nor are there studies that showed bite force reference values for this type of sample.

Therefore, studies aimed at understanding the functional alterations in patients with spinocerebellar ataxia serve the purpose of demonstrating to health professionals that the stomatognathic system may be compromised, and that treatments directed to this complex system need to be carefully designed, with great caution, so that no more functional impairments occur.

This study had limitations in its design because the psychological stress of the patients was not analyzed, a factor which could influence the greater electromyographic activation of the group with spinocerebellar ataxia.

## Conclusions

Our findings suggest that the spinocerebellar ataxia modifies the function of the stomatognathic system, as observed by increased masticatory muscles activity and reduction of maximal molar bite force.
